# The Effect of Loneliness on Death Anxiety in the Elderly During the COVID-19 Pandemic

**DOI:** 10.1177/00302228211010587

**Published:** 2021-04-20

**Authors:** Türkan Akyol Guner, Zeynep Erdogan, Isa Demir

**Affiliations:** 1 Department of Social Work, Faculty of Health Sciences, Zonguldak Bulent Ecevit University, Turkey; 2 Nursing Department, Vocational School of Health Services, Bulent Ecevit University, Kozlu, Turkey; 3 Department of Social Work, Faculty of Health Sciences, Zonguldak Bulent Ecevit University, Turkey

**Keywords:** COVID-19, pandemic, elderly, loneliness, death anxiety

## Abstract

The aim of the study is to determine the effect on death anxiety of loneliness in the elderly during the COVID-19 pandemic. The population of this study that is descriptive and cross-sectional type consist of 354 elderly who meet the inclusion criteria from three different associations operating for charitable purposes in a city center located in north-west Turkey. The average score of Loneliness Scale of Elderly (LSE) of the elderly was determined as 11.39 ± 5.31, and the average score of Death Anxiety Scale (DAS) of the elderly was determined as 8.54 ± 4.82. According to these results, it was found that the elderly experienced acceptable levels of loneliness and moderate death anxiety. A statistically significant difference was found in the LSE and DAS scores of the elderly according to their age, marital status, education status, chronic illness status and living at home with relatives. In addition, during the COVID-19 epidemic, the scale scores of the elderly who have increased worries, who have a hobby at home, and who communicate with their relatives via social media/mobile phones were found to be statistically significant (p < 0.05).

Societies have witnessed various epidemics that caused serious health problems and deaths throughout history. Today, once again, the whole world is fighting the new COVID-19 virus, which is an invisible enemy, and is going through a very difficult time (Nadeem, 2020). Due to the high rate of spread and mortality of this virus, which causes serious acute respiratory system infections, strict isolation measures have been applied especially to those in risk groups in countries where the epidemic occurs (Karcıoğlu, 2020; Velavan & Meyer, 2020; Wang et al., 2020). In this process, individuals over the age of 65 and with chronic diseases were considered to be at high risk in terms of mortality due to the “immunosenescence”, which known as immunodeficiency developing due to aging and it was considered to be the first group to leave social environments in our country as a scientific step within the scope of isolation measures (Ministry of Health, 2020; World Health Organization, 2020).

It is known that the proportion of the elderly population is increasing all over the world and even in our country which has a younger population compared to most of the world countries. The proportion of people at or above 65 years among the general population in Turkey rose by 9.1% in 2020 compared to 8% in 2019 representing an increase of 21.9% (TSI Population Projections, 2023–2080). Considering that 95% of deaths due to COVID-19 are over the age of 60, the mortality rate reaches 15% in the elderly and that 8 out of 10 deaths occur in the elderly with at least one chronic disease (such as cardiovascular disease, hypertension and diabetes), it becomes even more important that the elderly population ratio is affected minimally by the pandemic (European Centre for Disease Prevention and Control, 2020; World Health Organization, 2020). For all these reasons, a rule of social isolation and reducing physical contact was introduced in order to protect elderly in this highly contagious disease, and it was requested to limit the visits of family members or friends to elderly (Oflaz, 2020).

It has been determined that more than 17% of the population over the age of 65 in the world experience loneliness and social isolation without any isolation (quarantine or deliberate isolation) even under normal conditions, and 43% feel alone(http://www.euro.who.int/en/health-topics/healthemergencies/coronavirus-covid-19/news/news/2020/3/mental-health-and-psychological-resilience-during-thecovid-19-pandemic; Yılmaz & Akyazıcı, 2020). It has been reported that with mandatory quarantine and social isolation, such as the COVID-19 process, the elderly can experience an alarming feeling of loneliness(see https://www.apa.org/news/apa/2020/03/covid‐19‐danger‐physical‐health). While the feeling of loneliness is a subjective feeling of being alone, it causes many health problems, especially in elderly due to the forced loneliness periods and social isolation during the epidemic. It has been reported that the intense experience of this feeling increases the risk of heart disease, dementia, forgetfulness and other health problems, adversely affects the immune system and increases the risk of death equally with risk factors such as smoking, lack of physical activity, and obesity (https://www.cdc.gov/nchs/nvss/vsrr/COVID19/index.htm; https://www.nap.edu/catalog/25663/social-isolation-and-loneliness-in-older-adults-opportunities-for-the; Hall et al., 2008; Luanaigh & Lawlor, 2008; Sarvada, 2013; Yang et al., 2020). With this mandatory isolation applied during the COVID-19 pandemic, it was determined that the elderly people's death anxiety increased in addition to their physical health problems (Joanne & Debra, 2020; Santini et al., 2020; Torun, 2020; Türk, 2020).

Death anxiety has been called the fear that people feel in the face of the end of their existence in this world (Hick, 1990). People's perception of death is shaped according to the death events they see in their environment, the developmental characteristics of the age period and the personal experiences they develop depending on the religious and cultural values they believe (Hökelekli, 1991; Kurçer & Erdoğan, 2020). The prevalence of death anxiety in the elderly is controversial. In some studies, death anxiety was found to be high due to reasons such as physical problems, movement limitations and dependency on others (Assari & Lankarani, 2016; Bala & Maheshwari, 2018; Galt & Hayslip, 1998; Khademi et al., 2021; Meng et al., 2020; Suhail & Akram, 2002; Yao et al., 2020), while in others it was determined that this anxiety decreased due to acceptance of death with maturity developed by the age (Jamadar & Chawla, 2020; Oztürk et al., 2012).

As it is known that people experienced conditions such as sickness, loneliness, anxiety, panic, stigma and death anxiety in previous epidemic periods (Hall et al., 2008), it has been stated that individuals will be likely to experience similar problems in the COVID-19 epidemic (Delam & Izanloo, 2020; Hall et al., 2008; Qui et al., 2020; Torun, 2020; Wang et al., 2020).

For these reasons, the aim of this study is to determine the effect on death anxiety of loneliness in the elderly during the COVID-19 pandemic and to contribute to the initiatives that can be planned for the elderly according to the results of the research.

## Method

This descriptive and cross-sectional type study was conducted between 10.06.2020 and 15.10.2020 in three different associations operating for charitable purposes in a city center located in the north west of Turkey. While there were 392 elderly registered in associations, the study was completed with 354 elderly who met the inclusion criteria applied by the researchers, which were prepared with reference to similar studies. Before starting to collect data for the study, elderly were contacted by phone and the purpose, method and benefits of the study were explained. Inclusion criteria in the study; being 65 years and over, not having a hearing problem, being able to communicate verbally and being willing to participate in the study. The exclusion criteria are having any mental disorder diagnosed by the relevant physician, having diagnosed of depression by the relevant physician and taking depression medication during the study, having a fatal illness, having a communication problem. Due to the pandemic, the data were collected by phone calls and home visits for support purposes if requested.

### Data Collection Tools

**The data were collected using the “Socio-demographic data form”**: The Loneliness “Scale for the Elderly (LSE)” and the “Death Anxiety Scale (DAS)”. Diagnostic Form was prepared by the researchers.

**Socio-demographic data form:** It consists of 11 questions inquiring the socio-demographic characteristics of the elderly and their knowledge about the COVID-19 pandemic.

**LSE:** Developed by Gierveld and Kamphuis (1985) and revised in 1999, this scale consists of 11-item, 3-point likert-type (0 = yes, 1 = might, 2 = no), separated into two subspaces namely, emotional loneliness and social loneliness (Tilburg et al., 2004). According to the scores obtained from the scale; 0–4 points are evaluated as “not feeling lonely”, 5–14 points as “acceptable loneliness”, 15–18 points as “very lonely” and 19–22 points as “very intensely lonely”. Its Turkish validity and reliability were made by Akgül and Yeşilyaprak (2015), and the internal consistency coefficient was found to be α = 0.85, (Akgül&Yeşilyaprak, 2015), as a result of its use in our study, it was found to be α = 0.88.

**DAS:** The scale was developed by Templer (1970) and its validity and reliability study was performed by Şenol (1989) in our country (Templer, 1970; Şenol, 1989), it is a 15-item scale that measures the anxiety and fears of the individuals about their own death and answered as true-false. The sum of the scores obtained from the scale gives the death anxiety score, 0–4 points “mild”, 5–9 points “moderate”, 10–14 points “severe”, 15 points “panic level” death anxiety. In Senol's reliability study, the internal consistency coefficient of the scale was found to be α = 0.86, and as a result of its use in our study, it was found to be α = 0.89.

### Statistical Analysis

Statistical analysis of the data was performed using SPSS 22.0 (IBM Corporation, Armonk, NY, USA) package program. In data analysis, percentage, mean±SD, t-test, One-Way Analysis of Variance (ANOVA), Kruskall Wallis H Test, Tukey test, Mann Whitney U test and Pearson Correlation Coefficient were used to determine the differences in variables with more than two groups. Multiple regression analysis was used to determine the effects of independent variables on dependent variables. The significance level in the tests was taken as p < 0.05.

### Ethical Issues

In order to conduct the study, approval of the ethics committee was obtained from the Zonguldak Bülent Ecevit University Human Research Ethics Committee with the decision dated 27.04.2020/790 and then from the Ministry of Health with the form number 2020–06-09T16_56_07 to conduct the study during the pandemic period. Before the data was collected, the conditions of “Informed Consent” and “Volunteerism Principle” were fulfilled by informing the elderly about the purpose and benefits of the research.

## Results

The socio-demographic characteristics of the elderly are given in Table 1. The average age of the elderly people who participated in our study was 68.28 ± 2.90, 76.6% (n = 271) were male, 33.6% (n = 120) were primary school graduates, 87.0% (n = 308) were married, 44.1% live at home with their spouse and children and 71.5% (n = 253) of them had chronic diseases (Table 1).

**Table 1.** Demographic Characteristics of Elderly (N = 354).

Age (years) Mean ± SD	68.28 ± 2.90	N	%
Gender	Female	83	23.4
	Male	271	76.6
Education status	Primary school	120	33.9
	Middle school	68	19.2
	High school	106	29.9
	University	60	16.9
Marital status	Married	308	87.0
	Single	46	13.0
Living together at home	Alone	16	4.5
	Spouse	152	42.9
	Spouse/Child	156	44.1
	Other	30	8.5
Chronic disease	Yes	254	71.5
	No	101	28.5

When the findings of the elderly participating in the study regarding the pandemic period are examined; 45.5% (n = 161) had sufficient knowledge about COVID-19 according to their own assessment, 49.2% (n = 174) did not have any hobby at home during this period, 69.2% (n = 245) communicated with their relatives via social media and mobile phones, 51.1% of them (n = 181) increased levels of worry according to the changing emotional and social status during this process and 74.9% (n = 265) increased in feelings of spirituality in the COVID-19 (Table 2).

**Table 2.** Findings of the Elderly About the Pandemic Period (N = 354).

	N	%
Sufficient knowledge about COVID-19		
Yes	161	45.5
No	16	4.5
Partly	177	50
Diagnosis of COVID-19 in the family		
Yes	48	13.6
No	306	86.4
Dealing with hobby at home during the COVID-19		
Yes	114	32.2
No	174	49.2
Communicating with social media/mobile phone in the COVID-19		
Partly	66	18.6
Yes	245	69.2
No	25	7.1
Partly	84	23.7
Increase in worry in the COVID-19		
Yes	181	51.1
No	15	4.2
Partly	158	44.6
Increase in feelings of spirituality in the COVID-19		
Yes	80	22.6
No	9	2.5
Partly	265	74.9

The average LSE score of elderly who participated in the study was determined as 11.39 ± 5.31, and it was determined that they experienced acceptable level of loneliness according to the scale evaluation. In addition, the average score of the Emotional Loneliness sub-dimension, which is one of the LSE sub-dimensions of the elderly, was 6.99 ± 2.48, and the average score for the sub-dimension of Social Loneliness was 4.40 ± 3.12, and it can be said that both of them are at a medium level. The average DAS score of the elderly was determined as 8.54 ± 4.82 and according to the scale evaluation, it was found that the death anxiety experienced by the elderly was at a moderate level (Table 3).

**Table 3.** The Distribution of LSE Scores of the Elderly.

Scales	N	Mean+SD	Min-Max
LSE	354	11.39+5.31	0–22
*Emotional Loneliness	354	6.99+2.48	0–12
*Social Loneliness	354	4.75+3.12	0–10
DAS	354	8.54+4.82	0–15

LSE: Loneliness Scale for Elderly, *LSE sub-dimensions, DAS: Death Anxiety Scale.

Table 4 shows the comparison of scale score averages according to the socio-demographic characteristics of elderly. It was observed that there was no statistically significant difference between LSE and DAS scores with sub-dimensions according to the gender of the elderly (p > 0.05), and a statistically significant difference was found in scale scores according to marital status and education level (p < 0.05).It was observed that those who stated their marital status as single had higher LSE general and sub-dimension scores and the mean DAS score than those who stated their marital status as married (p = 0.000).According to the education level, a significant difference was determined according to the LSE (p = 0.029) and the sub-dimensions (p = 0.032, p = 0.026) and the mean DAS score (p = 0.038) of the primary school graduates. It was determined that the chronic disease status of the elderly significantly increased the LSE (p = 0.017) and its sub-dimensions (p = 0.000, p = 0.019) and the mean DAS scores (p = 0.000).

**Table 4.** The Distribution of LSE Scores According to the Sociodemographic Characteristics.

Variable		N	Emotional** Loneliness	Social** Loneliness	LSE	DAS
Gender^a^	Female	83	6.75+2.75	3.95+3.64	10.71+6.21	8.67+5.36
	Male	271	7.06+2.39	4.54+2.94	11.60+5.00	8.50+4.65
			t=−0.985; p = 0.325	t = −0.579; p = 0.132	t = −1.348; p = 0.178	t = 0.285; p = 0.77
Marital status^a^	Married	308	6.78+2.45	4.06+3.05	10.84+5.19	8.05+4.67
	Single	46	8.41+2.21	6.69+2.60	15.10+4.62	11.82+4.60
			t = −4.288; p = 0.000*	t=−5.550; p = 0.000*	t = −5.265; p = 0.000*	t = -5.122; p = 0.000*
Spouse (2)	152	7.55+2.22	5.17+2.67	12.73+4.49	9.96+3.67
Spouse/child (3)	156	6.03+2.44	2.97+3.02	9.00+5.18	6.18+4.79
Otder (4)	30	7.96+2.07	6.10+2.41	14.06+4.23	10.96+4.81
		*X*^2^ = 18.713; p = 0.000*	*X*^2^ = 28.732; p = 0.000*	*X*^2^ = 27.271; p = 0.000*	*X*^2^ = 31.203; p = 0.000*
Differences		1 > 2; 1 > 3; 2 > 3; 4 > 3	1 > 2;1 > 3;2 > 3;4 > 3	1 > 2;1 > 3;2 > 3;4 > 3	1 > 2; 1 > 3; 2 > 3; 4 > 3
Education status^b^	PrimarySchool (1)	120	7.90+2.30	5.59+2.79	13.49+4.88	10.96+3.86
	MiddleSchool (2)	68	6.86+2.53	3.76+3.09	10.63+5.45	7.13+4.95
	HighSchool (3)	106	6.50+2.26	3.63+3.04	10.13+4.84	7.06+4.75
	University (4)	60	6.20+2.64	3.11+3.28	10.31+5.62	7.90+4.75
			F = −9.436; p = 0.026*	F = −9.71; p = 0.032*	F = 10.259; p = 0.029*	F = 17.954; p = 0.038*
	**Differences**		1 > 2,3,4	1 > 2,3,4	1 > 2,3,4	1 > 2,3,4
Chronic disease^a^	Yes	253	7.70+2.22	5.39+2.69	13.10+4.53	10.13+4.21
	No	101	5.21+2.20	1.91+2.73	7.12+4.71	4.56+3.89
			t = −9.510; p = 0.019*	t = −10.96; p = 0.000*	t = −11.074; p = 0.017*	t = −11.470; p = 0.000*
Living together at home^c^	Alone (1)	16	9.25+2.29	7.81+2.66	17.06+4.02	13.43+3.79

LSE: Loneliness Scale for Elderly; DAS: Death Anxiety Scale.

^a^Independent groups t test.

^b^ANOVA.

^c^Kruskall Wallis H Test.

*p < 0.05.

**LSE sub-dimensions.

Significance of bold values is differences : Turkey test.

The comparison of the findings of the elderly regarding the pandemic according to the scale scores is given in Table 5. While there was no statistically significant difference between the state of having sufficient knowledge about COVID-19 and the mean scores of LSE and its sub-dimensions (p > 0.05), it was determined that there was a statistically significant difference with the mean score of the DAS scale (p = 0.012).It was determined that those who did not have any hobby at home during the COVID-19 process had higher scale scores and this situation was statistically significant (p = 0.000).

**Table 5.** Comparison of the Scale Scores According to the Findings of the Elderly About the Pandemic Period (N = 354).

		N	Emotional** Loneliness	Social** Loneliness	LSE	DAS
Sufficient knowledge about COVID-19^a^	Yes	161	4.42+2.05	4.31+2.62	6.73+4.27	5.68+4.27
	No	16	5.06+1.28	5.43+1.50	7.50+2.36	11.87+3.07
	Partly	177	5.23+2.06	5.03+2.36	7.26+4.08	10.83+3.94
	**Differences**		*X*^2^ = 123.15; p = 0.324	*X*^2^ = 136.90; p = 0.288	*X*^2^ = 149.62; p = 0.319	*X*^2^ = 99.36; p = 0.012^*^
						*2 > 1,3*
Diagnosis of COVID-19 in the family^b^	Yes	48	7.52+2.67	4.68+2.99	12.20+5.28	9.39+4.62
	No	306	6.91+2.44	4.35+3.14	11.27+5.31	8.40+4.84
			t = 1.581; p = 0.112	t = 0.67; p = 0.500	t = 1.13; p = 0.257	t = 1.319; p = 0.18
Dealing with hobby at home during the COVID-19^c^	Yes (1)	114	5.50+2.13	1.71+2.57	7.21+4.31	5.17+4.91
	No (2)	174	8.17+2.37	6.06+2.49	14.24+4.59	11.04+3.59
	Partly (3)	66	6.46+1.64	4.66+2.17	11.13+3.52	7.77+3.58
			F = 54.11; p = 0.000^*^	F = 107.89; p = 0.000^*^	F = 91.22; p = 0.000^*^	F = 73.10; p = 0.000^*^
	**Differences**		**2 > 1,3; 3 > 1**	**2 > 1,3; 3 > 1**	**2 > 1,3; 3 > 1**	**2 > 1,3; 3 > 1**
Communicating with social media / mobile phone in the COVID-19^a^	Yes (1)	245	6.24+2.29	3.26+2.93	9.51+4.87	7.14+4.80
	No (2)	25	9.84+1.24	7.84+1.62	17.68+2.67	12.44+2.80
	Partly (3)	84	8.32+2.12	6.71+1.66	15.03+3.55	11.46+3.13
			*X*^2^ = 77.10; p = 0.021^*^	*X*^2^ = 114.94; p = 0.019^*^	*X*^2^ = 102.10; p = 0.011^*^	*X*^2^ = 68.01; p = 0.013^*^
	**Differences**		**2 > 3,1; 3 > 1**	**2 > 1; 3 > 1**	**2 > 3,1; 3 > 1**	**2 > 1; 3 > 1**
Increase in worry in the COVID-19^a^	Yes (1)	181	8.31+2.10	5.90+2.56	14.21+4.22	11.06+4.26
	No (2)	15	4.80+1.82	1.20+2.48	6.00+4.20	4.33+4.99
	Partly (3)	158	5.68+2.08	2.99+2.88	8.68+4.69	6.05+3.73
			*X*^2^ = 112.65; p = 0.000^*^	*X*^2^ = 90.43; p = 0.000^*^	*X*^2^ = 120.26; p = 0.000^*^	*X*^2^ = 102.26; p = 0.000^*^
	**Differences**		**1 > 3,2**	**1 > 3,2; 3 > 2**	**1 > 3,2**	**1 > 3,2**
Increase in feelings of spirituality in the COVID-19^a^	Yes (1)	80	7.65+2.53	4.78+3.09	12.43+5.41	9.77+5.14
	No (2)	9	8.33+1.32	5.66+3.04	13.00+4.33	7.00+3.12
	Partly (3)	265	6.75+2.45	4.24+3.13	10.99+5.26	8.12+4.71
			*X*^2^ = 10.95; p = 0.031^*^	*X*^2^ = 2.78; p = 0.247	*X*^2^ = 6.10; p = 0.291	*X*^2^ = 9.47; p = 0.000^*^
	**Differences**					**1 > 3**

LSE: Loneliness Scale for Elderly, DAS: Death Anxiety Scale.

^a^Kruskall Wallis H Test.

^b^Independent groups t test.

^c^Anova.

*p<0.05.

**LSE sub-dimensions.

Significance of bold values is differences : Turkey test

In this process, it was observed that those who did not communicate with social media or mobile phones had higher and statistically significant scores on the LSE (p = 0.011), sub-dimensions (p = 0.021; p = 0.019) and DAS (p = 0.013) scale scores. In the elderly who participated in the study, it was determined that there was a statistically significant difference between the increased anxiety during the COVID-19 and the scale scores (p = 0.000), it was found that the scale scores of the elderly with increased anxiety were higher. When the effect of the sense of spirituality experienced by the elderly on the COVID-19 was examined, it was determined that the DAS score of the elderly who stated that their sense of spirituality developed or partially developed was statistically significant (p = 0.000). No significant difference was found between the scale scores and the coronavirus diagnosis in the family (p > 0.05).

In Table 6, it was determined that there is a high and significant positive correlation between the sense of loneliness and death anxiety experienced by the elderly (p < 0.01; r: 0.77). In addition, positive, high and significant relationships were found between death anxiety experienced by the elderly and emotional loneliness (p < 0.01; r:0.73) and social loneliness (p < 0.01; r: 0.72). By looking at these correlation values, it can be said that as the emotional and social loneliness levels of the elderly increase, a significant increase in death anxiety may occur. A positive, moderate and significant correlation was found between the age of the elderly and their loneliness and death anxiety scores. According to this, it is seen that the feeling of loneliness and death anxiety increase as the age increases.

**Table 6.** Correlation of Elderly Between Loneliness, Death Anxiety and Age (N: 354).

	**LSE**	EmotionalLoneliness	Social Loneliness	**DAS**	**Age**
**LSE**	1.00				
Emotional Loneliness	0.90*	1.00			
Social Loneliness	0.91*	0.79*	1.00		
**DAS**	0.77*	0.73*	0.72*	1.00	
**Age**	0.44*	0.36*	0.46*	0.38*	1.00

Pearson moments multiplication correlation.

*p<0.01.

In the regression table made to explain the effect of the level of loneliness experienced by the elderly on death anxiety, it is seen that emotional loneliness and social loneliness are significant predictors of death anxiety (F = 248.266; p = 0.00), it is also seen that death anxiety is positively affected by the increase of emotional and social loneliness (Table 7).

**Table 7.** Regression Table of the Effect on Death Anxiety of Loneliness in Elderly (N: 354).

Dependent variable	Independent variable	β	t	p	R^2^	Adj. R^2^	F
Emotional Loneliness	0.725	19.731	0.00*			
Emotional Loneliness	0.407	7.258	0.00*			
Social Loneliness	0.402	7.172	0.00*			
Model 1Death Anxiety	Constant		−2.449	0.01*	0.525	0.524	389.319
Model 2Death Anxiety	Constant		0.527	0.00*	0.586	0.583	248.266

*p: 0.00; R = regression coefficient.

## Discussion

Elderly who deal with health problems, often have difficulties in meeting their personal needs and worry about their future, experience intense feelings of loneliness, uncertainty and anxiety, which are the most important psychological consequences of social isolation during the pandemic (Galea et al., 2020; Steinman et al., 2020). Studies have reported that approximately one-third of the elderly often or always experience loneliness and social isolation (Jansson et al., 2018; Savikko et al., 2005). It is thought that it is very important to diagnose the risks and effects these emotions may cause on the person. The fact that elderly loneliness, which was an important public health problem even before the pandemic, increased with the social isolation experienced during pandemic measures and the social distance rule has been a primary concern by scientists (Armitage & Nellums, 2020; Ayalon et al., 2021; Brooke & Jackson, 2020).

Loneliness is known to negatively affect people's physical and psychological well-being and increase death anxiety, especially in the elderly (Birgit et al., 2018; Cohen-Mansfield et al., 2016; Valtorta et al., 2016). However, while it is not known exactly how this pandemic affects loneliness and death anxiety, it has been reported that new researches are required to determine the negative effects such as loneliness that may occur especially in the elderly and its accompanying anxiety and death anxiety (Delam & Izanloo, 2020; Khademi et al., 2021; Menzies & Menzies, 2020; Pasion et al., 2020). It is thought that this study, which was planned for this purpose, will contribute to the literature in terms of originality since it is the first study to examine the feeling of loneliness and death anxiety experienced by the elderly during the COVID-19 pandemic and the factors affecting these two conditions.

Being a multidimensional concept, loneliness is defined as a universal situation that every individual can live in a certain period of their lives, regardless of age, gender, race and class, and it is reported that it is felt most in old age (Ponizovsky&Ritsner, 2004; Oz, 2010). Although the social isolation process applied to the elderly due to their bio-psychosocial deficits is an important strategy to fight COVID-19, it has been reported to be an important cause of depression, anxiety disorders and loneliness (see https://www.cdc.gov/coronavirus/2019-ncov/prepare/managing-stress-anxiety.html). Older people feel emotionally and socially alone as a result of the social isolation they experience, as if they are in emptiness, socially inadequate, dependent on someone and not accepted by people (Kurt et al., 2010; Yerli, 2017). In emotional loneliness, the individual has lost close relationships and feels as if he is in emptiness while in social loneliness, the individual is unable to gain a place of acceptance in the society, sees himself as incomplete and thinks that his position in the society has regressed in terms of quantity and quality. In this study, general loneliness and emotional and social loneliness levels experienced by elderly during the COVID-19 pandemic were examined, and it was determined that the elderly experienced moderate loneliness and social and emotional loneliness. In a study examining the effect of the pandemic on the sense of loneliness experienced by the elderly, it was determined that the elderly experienced moderate loneliness. Again, as a result of a study conducted with 1679 elderly Dutch adults aged 65 to 102 years to evaluate loneliness and mental health in the elderly during the COVID-19, it has been determined that the feeling of loneliness increased as a result of social isolation, loss and concerns about the epidemic and especially emotional health problems increases even more than social loneliness (Van Tilburg et al., 2020). Similarly, in a study conducted in the United States to determine the sense of loneliness experienced by people during the pandemic, it was determined that of loneliness was experienced at a rate of 51% and emotional loneliness was more intense, especially at the age of 65 and over (Luchetti et al., 2020).

When the feeling of loneliness is compared with the socio-demographic characteristics of the elderly; it was determined that there are statistically significant differences in loneliness level according to age, marital status and educational status, whereas gender does not affect loneliness. It has been reported that the most important socio-demographic feature affecting the feeling of loneliness in the elderly is age (Sinoff, 2017), and similar studies conducted during the pandemic also found that loneliness levels increased with age (Heidinger & Richter, 2020; Jamadar & Chawla, 2020; Pasion et al., 2020). As a result of this study, it was found that there was a positive, moderate and significant relationship between the age of the elderly and the sense of loneliness, and similar to the literature, the feeling of loneliness increased with increasing age. In a study examining the effect of the COVID-19 pandemic on the feeling of loneliness that may be experienced in the elderly, which is conducted similar to our study, it was reported that the determinants of loneliness were age, gender, marital status, educational status, people living together and social support. In addition, it was determined that the sense of loneliness of the elderly living alone increased statistically significantly compared to the pre-pandemic, and it was found that the coping mechanisms of the elderly living with even one person were stronger and the feeling of loneliness was felt less. Similarly, in a study that evaluated the effects of the pandemic on loneliness in the elderly and including a total of 1990 people in four groups and including January-May, women, those living alone, those who do not communicate with their neighbors or friends experienced more loneliness. It was also found that the feeling of loneliness increased further as the period of social isolation extended. In an online study conducted with 2074 people between the ages of 50–80 regarding who mostly experienced the feeling of loneliness experienced during the pandemic, it was reported that the feeling of loneliness was more intense in women, those who did not communicate with their relatives, those who lived alone and those with health problems (Piette et al., 2020). In a study conducted on the determinants of loneliness in the adult group in the COVID-19 pandemic in the United States, similar to our findings, it was found that loneliness increased significantly in those with any mental health problems, those living alone, and those without social support (Bu et al., 2020). These results are similar to our study results except gender. Similar to our findings, no significant relationship was found between the sense of loneliness and gender in the elderly in the study by Kapıkıran et al. (2016). In similar studies conducted to determine the loneliness levels of the elderly before the pandemic, in parallel with our findings, it was determined that as the age increases, the levels of loneliness are higher in those who stated their marital status as single, those with a low education level, those living alone at home, and the elderly with chronic diseases (Aslantaş et al., 2015; Cohen-Mansfield & Perach, 2015; Jakobsson & Hallberg, 2005; Khorshid et al., 2004; Lee et al., 2019; Routasalo et al., 2006; Savikko et al., 2005).

In our study, factors that may affect the level of loneliness related to the COVID-19 pandemic were also examined. According to this; in this process, it was determined that those who do not have any hobby in the home environment, do not communicate with their relatives via social media or mobile phone, and who have high anxiety due to the process, have higher sense of loneliness and the difference is statistically significant. In order to support elderly people during the social isolation period through the current pandemic and to ensure that their social, physical and psychological health needs are met, the elderly should be regularly called by their relatives, as well as regular visits by voluntary health and social organizations to minimize loneliness and the negative effects of loneliness are reported to be necessary (Joanne & Debra, 2020). In a report published on the loneliness of the elderly during the pandemic, it was stated that the elderly felt less lonely by communicating with their relatives or friends via social media or spending time together, even for a limited time each week (Piette et al., 2020). In a study aimed at reducing the loneliness of the elderly, it was found that when elderly people acquire a hobby for themselves, are supported socially, are active in social networks and communicate with their relatives through social media or online technologies, potentially their feelings of loneliness decrease (Käll et al., 2020). According to the findings obtained from these studies, it is necessary to increase the effective use of communication technologies in order to ensure communication and interaction among the elderly, and to support the elderly in gaining various hobbies.

Taking those who have positive coronavirus test into social isolation causes suicidal behavior and psychotic disorders in individuals with previous serious mental illness, while it causes loneliness associated with depression in those without mental illness (Krupp et al., 2020). In our study, no significant difference was found in the loneliness scores of those who had coronavirus diagnoses in their family or close environment and who were informed about the coronavirus. This result may be due to differences in the sample group.

Situations such as old age, illness, and loneliness make individuals anxious about the idea of death and may cause an increase in death anxiety (Babacan & Duman, 2017; Yelboğa, 2017). Death anxiety is one of the main problems of the aging process, and in fact, human beings have struggled with the feeling of death anxiety in various epidemics throughout history, and with the results obtained, it has been seen that death anxiety also causes a series of physical and psychological health problems in such pandemic and epidemic processes (Arpacı et al., 2011; Menzies & Menzies, 2020). During the pandemic we live in, it has been revealed that the item “I fear losing my life due to COVID-19” has the highest factor load in the scale developed to evaluate the fear of death, and it has been seen how important this fear and anxiety is for the elderly (Ahorsu et al., 2020). It was determined that the death anxiety experienced by the elderly participating in our study during the COVID-19 pandemic was above the medium level. According to data obtained from 1210 people living in China, it has been reported that the most important cause of psychological distress experienced during the COVID-19 is death anxiety (Wang et al., 2020). Similarly, studies on death anxiety experienced by the elderly during the COVID-19 have also found that death anxiety is high in individuals during this process, and the anxiety about their health and the psychological distress they experience increase death anxiety (Newton-John et al., 2020; Yao et al., 2020). In fact, death anxiety is among normal human behaviors. It affects human life even under normal conditions and causes various health problems. During the COVID-19 pandemic, it is predicted that people may unwittingly experience more fear and anxiety about death than ever before (Menzies & Menzies, 2020). Even in similar studies conducted before the pandemic, it was determined that the elderly experienced serious death anxiety (Cınar, 2016; Gündoğan, 2020; Köftegül, 2018 Ustüner-Top et al., 2010).

In this study, the relationship between death anxiety and socio-demographic characteristics of the elderly was evaluated. Death Anxiety Scale scores of those who reported marital status as single, those who graduated from primary school, those who lived alone at home, and the elderly with chronic diseases were found to be statistically significant and high. No significant differences were found by gender. A study examining the relationship between death anxiety and loneliness in the elderly found that increased age and low levels of education increased death anxiety. Also, contrary to our study, it was found that gender affects death anxiety, and women experience higher death anxiety than men (Gündoğan, 2020). In a study conducted in the pandemic of COVID-19, it was reported that those who were unmarried and living alone, especially those with a chronic respiratory system disease, experienced death anxiety more seriously (Leung et al., 2020).

The effects of pandemic findings on death anxiety have also been studied. Elderly who did not have sufficient knowledge of the pandemic, did not have a hobby at home, did not communicate with relatives, and had high worry due to the pandemic were found to have higher death anxiety. A study with elderly living in two different nursing homes during the pandemic was examined. In this study, it was determined that elderly had increased levels of worry and stress because they could not meet their families face-to-face due to the process of social isolation, and that they experienced serious death anxiety (Shi Yin, 2020). In our study, it was also determined that the emotions of spirituality developed in the elderly during the pandemic and that death anxiety increased significantly. It is known that one of the most valid tools that can be used for the elderly who try to interpret and make sense of what happened during the pandemic is spirituality (Gencer, 2019). Elderly generally think of spirituality to overcome loneliness, stress, depression, death anxiety and similar problems and to cope with difficulties (Klavuz & Klavuz, 2016). It is thought that elderly derive emotional support from their spiritual inclinations in coping with the mentioned psychological negativities and this way it is easier for them to cling to life.

Loneliness is a complex and often unpleasant emotion that is associated with physical problems as well as psychological problems such as anxiety and death anxiety. In our study, it was found that there is a positive, high and significant relationship between general loneliness, social-emotional loneliness and death anxiety of elderly during the COVID-19 pandemic. Similar to our study, a positive and significant correlation was found between psychological distress such as loneliness and anxiety and death anxiety in a study conducted during the pandemic that investigated the causes of death anxiety (Newton-John et al., 2020). In a similar study by Jamadar and Chawla (2020), a positive and significant relationship was found between loneliness and death anxiety in the elderly (Jamadar & Chawla, 2020). In another study, it was observed that there was a positive correlation between death anxiety and loneliness, depression and anxiety, and it was determined that as psychological problems increased, death anxiety increased (Menzies et al., 2019). In a study examining the factors that affect death anxiety among the elderly, a positive relationship was observed between loneliness and death anxiety (Kim et al., 2014).

## Conclusion and Recommendations

This study was conducted with the aim of examining the effect on death anxiety of loneliness experienced by elderly during the COVID-19 pandemic. A significant difference was found between death anxiety and feeling of loneliness among the elderly. In addition, age, marital status, educational status, living with relatives at home, chronic illness and some findings related to pandemic process were found to increase the feeling of loneliness and death anxiety in the elderly. Data from this study and other studies give the idea that death anxiety can be reduced by eliminating a sense of loneliness. For this, measures should be taken to eliminate the disadvantages created by socio-demographic characteristics.

The COVID-19 pandemic reminds that elderly in the vulnerable group should be strengthened and supported against emotions such as loneliness, social isolation, worry due to being alone, and death anxiety. In this sense, improving elderly' individual skill levels, increasing their use of technology and expanding their communication networks is extremely important to prevent them from feeling lonely. For this reason, it is very important to chat with elderly, to consult on health-related issues, to make video phone calls with relatives so that they do not feel alone. In addition, elderly people who are thought to be in need should be supported with cognitive behavioral therapies aimed at reducing loneliness. Voluntary organizations, nurses working in the field of public health and municipalities can develop comprehensive networks to support elderly and provide social contact with them. Being healthy and living is everyone's right. The pandemic has once again demonstrated this.

### Key Points

- COVID-19 is a pandemic with global health threat.
- Elderly are most vulnerable for severity and mortality.
- Elderly are most susceptible to mental health problems related to such pandemics.
- Special care needs to be taken for geriatric mental health during such crisis.
